# Mild Behavioral Impairment in Psychogeriatric Patients: Clinical Features and Psychopathology Severity

**DOI:** 10.3390/jcm12165423

**Published:** 2023-08-21

**Authors:** Camilla Elefante, Giulio Emilio Brancati, Zahinoor Ismail, Sara Ricciardulli, Maria Francesca Beatino, Vittoria Lepri, Antonella Famà, Elisabetta Ferrari, Linda Giampietri, Filippo Baldacci, Roberto Ceravolo, Icro Maremmani, Lorenzo Lattanzi, Giulio Perugi

**Affiliations:** 1 Psychiatry Unit, Department of Clinical and Experimental Medicine, University of Pisa, 56126 Pisa, Italy; camilla.elefante@phd.unipi.it (C.E.); giuliobrancati@gmail.com (G.E.B.); sara.ricciardulli@yahoo.com (S.R.); mariaf.beatino@gmail.com (M.F.B.); vittoria.lepri@gmail.com (V.L.); giulio.perugi@med.unipi.it (G.P.); 2 Departments of Psychiatry, Clinical Neurosciences, Community Health Sciences, and Pathology and Laboratory Medicine, Hotchkiss Brain Institute & O’Brien Institute for Public Health, University of Calgary, Calgary, AB T2N 1N4, Canada; ismailz@ucalgary.ca; 3 College of Health and Medicine, University of Exeter, Exeter EX4 4QG, UK; 4 Psychiatry Unit, Azienda Ospedaliero-Universitaria Pisana, 56126 Pisa, Italy; antonellafama@hotmail.com (A.F.); lizzi59@hotmail.it (E.F.); llattanzi@blu.it (L.L.); 5 Neurology Unit, Santa Chiara University Hospital, 56126 Pisa, Italy; lindagiampietri@gmail.com (L.G.); filippo.baldacci@unipi.it (F.B.); robertoceravolo66@gmail.com (R.C.); 6 G. De Lisio Institute of Behavioral Sciences, 56127 Pisa, Italy; 7 Addiction Medicine, Saint Camillus International University of Health and Medical Sciences (UniCamillus), 00131 Rome, Italy

**Keywords:** Mild Behavioral Impairment (MBI), apathy, aging, cognitive impairment, dementia

## Abstract

The Mild Behavioral Impairment (MBI) concept was developed to determine whether late-onset persistent neuropsychiatric symptoms (NPSs) may be early manifestations of cognitive decline. Our study aims to investigate the prevalence and differentiating features of MBI with respect to major neurocognitive disorders (MNDs) and primary psychiatric disorders (PPDs). A total of 144 elderly patients who were referred to our psychogeriatric outpatient service were recruited. The severity of mental illness was evaluated by means of the Clinical Global Impression Severity scale, the severity of psychopathology was evaluated by means of the Brief Psychiatric Rating Scale (BPRS), and overall functioning was evaluated by means of the Global Assessment of Functioning scale. The sample included 73 (50.6%) patients with PPDs, 40 (27.8%) patients with MBI, and 31 (21.5%) patients with MNDs. Patients with MNDs reported the greatest severity of mental illness, the highest BPRS Total, Psychosis, Activation, and Negative Symptom scores, and the lowest functioning. Patients with MBI and PPDs had comparable levels of severity of mental illness and overall functioning, but MBI patients reported higher BPRS Total and Negative Symptom scores than PPD patients. Patients with MBI frequently reported specific clinical features, including a higher severity of apathy and motor retardation. These features merit further investigation since they may help the differential diagnosis between MBI and PPDs.

## 1. Introduction

The prevalence of cognitive impairment and dementia is rising as the world’s population ages [1,2,3,4,5,6,7,8,9]. To maximize the success rate of dementia clinical trials, it is crucial to improve the recruitment of subjects who are still in the pre-symptomatic phases of cognitive decline but are likely to develop major neurocognitive disorders (MNDs) in the future [10,11,12,13]. Disease-modifying therapies, in fact, are mostly focused on early stages of cognitive decline [14,15,16]. Although the current research has focused on identifying risk factors for dementia, determining whether clinical signs and symptoms are prodromal to cognitive deterioration remains difficult.

Psychiatric disorders, especially depression, could be early manifestations of dementia in older people [17,18,19]. For instance, some studies revealed that the age at which depression first manifests will influence its association with dementia. Indeed, late-onset depressive symptoms, though not early-onset depression, have been considered as a risk factor for developing dementia or Alzheimer’s disease (AD) [20,21,22,23].

More recently, several studies have demonstrated that the onset of neuropsychiatric symptoms (NPSs) late in life is associated with a greater incidence of Mild Cognitive Impairment (MCI) and dementia [24,25,26,27]. Since NPSs are frequent in the prodromal and preclinical phases of dementia and may be non-invasively and inexpensively identified, they are an ideal candidate for large-scale screening of subjects at risk for dementia. Nevertheless, a variety of limitations prevent definitive conclusions to be drawn. In particular, the timing between the onset of NPSs and the development of cognitive impairment, as well as the age at which NPSs first appear, have not always been explored.

The construct of Mild Behavioral Impairment (MBI) has been outlined by a working group from the Alzheimer’s Association to allow the systematic evaluation of the emergence and course of NPSs in dementia-free individuals [28]. MBI criteria describe the emergence of meaningful and persistent NPSs, occurring from the age of 50, representing a change from the individual’s usual behavior or personality. MBI symptoms are grouped into five domains evaluating apathy, mood/anxiety dysregulation, impulse dyscontrol, social inappropriateness, and psychosis. MBI can co-occur with or even precede MCI, while the diagnosis of dementia is considered exclusionary. On the basis of MBI criteria, a specific instrument to assess and rate severity of NPSs, the MBI-Checklist (MBI-C), was developed and validated [29].

Based on the existing literature, the prevalence of MBI varies depending on the populations taken into account, the recruitment setting, and the evaluation tool adopted. According to studies performed in memory clinics employing the MBI-C, approximately one-third of Subjective Cognitive Disorder patients and half of MCI patients can be diagnosed with MBI [29,30,31]. In patients without considerable cognitive impairment, the prevalence of MBI instead appears to be lower. For instance, MBI is present in only 6.2–10% of volunteers recruited online [32,33].

Long-term studies on cognitively normal patients and patients with MCI have shown that MBI symptoms are associated with significantly greater rates of incident cognitive decline [34]. In particular, the presence of MBI was demonstrated to be a predictor of clinical and neuropathological progression to AD in a sample of cognitively normal individuals [35]. Specifically, psychotic symptoms had the greatest impact on the risk of progression to AD [35], a finding recently validated in cognitively normal patients and MCI [36]. In another one-year follow-up study, cognitively normal older adults with MBI developed substantial deficits in attention and working memory compared to those without [32]. Additionally, indicators of dementia such as beta-amyloid, tau, neurofilament light, and brain atrophy have all been related to the presence of MBI in cognitively normal individuals [37,38,39].

By concentrating on NPSs that emerge later in life in patients without dementia, MBI has provided a framework to better understand the epidemiological and temporal association among onset of NPSs, impairment of functioning, and incident neurodegeneration. MBI-C attempted to distinguish between MBI-associated NPSs and NPSs related to psychiatric disorders of late life. As per MBI criteria, NPSs cannot be part of a formal mental disorder diagnosis, standing out as distinct from chronic psychiatric symptomatology as well as from recurring mental illness with late-life relapse. Nonetheless, it is conceivable that some patients have, at the same time, MBI and psychiatric disorders meeting traditional diagnostic criteria.

In a previous study, we showed that major depression and MBI can co-occur in late life. Interestingly, depressed elderly individuals with a comorbid diagnosis of MBI had similar severity and course of affective symptoms with respect to patients only diagnosed with geriatric depression. However, MBI was associated with a significantly lower improvement in functioning in the short term [40]. In other words, the presence of MBI in patients with late-life psychiatric disorders may have a detrimental effect on global functioning over time, consistent with the hypothesis that MBI may be the initial manifestation of neurodegenerative disease. Differences between MBI domains may also help explain this finding. Even in the context of historical depression, a change in presentation or type of depressive symptoms or onset of NPSs in a different domain may represent neurodegenerative disease and potentially qualify as MBI.

The detection of MBI may, thus, be helpful for prognostic purposes, not only in memory clinics but also in psychogeriatric settings. Matsuoka and colleagues retrospectively assessed the prevalence of MBI in a large sample of psychiatric outpatients over 50 years using diagnostic criteria developed by the International Society to Advance Alzheimer’s Research and Treatment (ISTAART) [41]. A low prevalence of MBI of 3.5% was found by the authors. However, while in that study, MBI criteria have been retrospectively applied based on chart reviews, our research is the first to investigate the prevalence of MBI in psychiatric patients by means of a specific interview, the MBI-C. Moreover, to the best of our knowledge, this is also the first study focused on clinical differences between the elderly with MBI and those with primary psychiatric disorders (PPDs) without co-existing NPSs.

The aim of this study was to systematically evaluate the occurrence of MBI in a sample of aged individuals (mean age = 74.67 ± 8.07) who were referred to our psychogeriatric outpatient service for mental health care. On the one hand, in order to further evaluate the MBI construct, patients with MBI were compared with patients diagnosed with MNDs. On the other, to identify potential hallmarks useful for diagnostic purposes, the differences with psychiatric patients that did not satisfy the diagnosis of MBI were assessed.

## 2. Materials and Methods

### 2.1. Participants

Participants were enrolled from patients attending the psychogeriatric outpatient service of Psychiatry Unit 2 at Pisa University Hospital (Italy) between June 2020 and May 2022, according to the following inclusion criteria: (1) age ≥ 50 years; (2) diagnosis of any psychiatric disorder according to the criteria of the Diagnostic and Statistical Manual of Mental Disorders, 5th edition (DSM-5) [42]; (3) and/or diagnosis of MBI according to the criteria of the ISTAART [28]; or (4) diagnosis of MNDs in accordance with DSM-5 criteria [42]. Patients diagnosed with Parkinson’s disease or other neurodegenerative Parkinsonism were excluded. Additionally, subjects were also excluded from the research if their clinical history was incomplete or if they were visited in the absence of a family member/caregiver. Of the 191 subjects screened, after the exclusion of those with neurodegenerative Parkinsonism and those without a source of collateral history, 144 patients were included in the study. All subjects provided written, informed consent for data collection to be analyzed and presented anonymously in aggregate form. The study was carried out in accordance with the Declaration of Helsinki. The protocol was approved by the Ethical Committee of the University of Pisa (N. 22537_Perugi).

### 2.2. Assessment

All patients were evaluated by psychiatric trainees with at least two years of experience in the field of psychogeriatrics under senior psychiatrist supervision. Educational history, marital status, family history of psychiatric and neurodegenerative conditions, medical comorbidity (including MCI diagnosed based on Peterson criteria [43,44,45]), current and lifetime psychiatric conditions according to DSM-5 criteria [42], and course features were all investigated at baseline. The Clinical Global Impression Severity scale (CGI-S) [46] was used as a proxy of psychiatric illness severity according to the clinician’s judgment at baseline. The Brief Psychiatric Rating Scale, Extended Version (BPRS) [47] and the Global Assessment of Functioning (GAF) [48] were used, respectively, to rate symptom severity and functioning level. BPRS subscales were derived according to the model proposed by Velligan and colleagues [49]. Moreover, according to our usual clinical routine, the Mini-Mental State Examination (MMSE) [50] was also administered.

### 2.3. MBI Diagnosis

The diagnosis of MBI was formulated based on a multi-step protocol. First, the MBI-C interview was administered in person to the patient and the patient’s caregiver [51]. Patients with MBI could also be diagnosed with PPDs, but specific MBI features such as the age of onset, severity, and qualitative pattern of NPSs were emphasized during the interview. For example, in comparison to mental disorders identified by DSM-5, NPSs pertaining to MBI show distinct qualitative characteristics and a different course. Particularly, in accordance with the MBI criteria [28], NPSs were rated as present only if they represented a clear change from the patient’s usual behavior or personality, including during previous mood episodes (i.e., new-onset symptoms), had a persistent course (≥6 months), and did not occur before the age of 50 years. Thus, MBI criteria permit a distinction between DSM-5 mental disorders and late-onset psychiatric symptoms. As previously suggested [33,52], a cut-off of 6.5 (i.e., ≥7) was used to identify patients screening positive for the MBI diagnosis, that is, patients showing a high level of NPSs. MBI-C findings, clinical history and medical files of patients screening positive for MBI were then discussed with two senior psychiatrists (CE and LL) who re-examined the patient and provided a consensus diagnosis of MBI.

### 2.4. Statistical Analyses

Descriptive statistics were used to summarize demographic and clinical characteristics of the sample. Patients were subdivided into three groups: (1) patients without MBI and MNDs, i.e., diagnosed with PPDs; (2) patients with MBI, with or without formal psychiatric diagnoses; and (3) patients with MNDs, with or without formal psychiatric diagnoses. Demographic and clinical variables (i.e., the prevalence of lifetime mood and anxiety disorders, current mood state, age at onset of psychiatric and mood disorders, family history of psychiatric and neurodegenerative conditions, presence of leukoencephalopathy at neuroimaging, MCI, mental status, and psychopathology severity and functioning) were compared among the groups. Analysis of Variance (ANOVA or Kruskal–Wallis test), with post hoc Dunn test, was used for comparisons of continuous variables. Comparative analyses of categorical variables were conducted using Pearson’s chi-squared tests (or Fisher’s exact test) with pairwise Fisher’s exact test as post hoc analysis. False discovery rate correction (FDR) for multiple comparisons was applied to post hoc contrasts. The Shapiro–Wilk test was used for the normality check. A statistical significance threshold of *p* < 0.05 was set in the analyses. All the analyses were performed using R Statistical Software (R-4.3.1-arm64.pkg) (Foundation for Statistical Computing, Vienna, Austria).

## 3. Results

### 3.1. Sample Characteristics

A total of 144 elderly patients (≥50 years) were recruited. The sample consisted of 54 (37.5%) men and 90 (62.5%) women. The average age was 74.67 ± 8.07, ranging from 50 to 88 years. As for marital status, 85 (59.0%) patients were currently married, 10 (6.9%) had never been married, 5 (3.5%) were separated/divorced, and 44 (30.6%) were widowed. Education history was available for 140 subjects: 6 (4.3%) patients had not completed elementary education, 49 (35.0%) had completed elementary education, 39 (27.9%) had completed middle education, 24 (17.1%) had a high school diploma, and 22 (15.7%) had higher education.

According to DSM-5 criteria, 112 (77.8%) patients had a diagnosis of mood disorder. Major depressive disorders were diagnosed in 37 (23.1%) subjects, 23 (16.0%) with a single episode and 14 (9.7%) with recurrent episodes. Bipolar spectrum disorders were observed in 75 (52.1%) cases: 24 (16.7%) subjects were diagnosed with bipolar disorder type 1, 28 (19.4%) with bipolar disorder type 2, 11 (7.6%) with cyclothymic disorder, with or without a history of major depressive episodes, and 12 (8.3%) with not otherwise specified bipolar disorders. Finally, 32 (22.2%) patients had no lifetime mood disorders. Overall, 81 (56.3%) patients were diagnosed with at least one lifetime anxiety disorder, the most common being panic disorder and generalized anxiety disorder, respectively diagnosed in 56 (38.9%) and 41 (28.5%) patients.

Over one-half of patients were euthymic at the moment of the assessment (N = 82, 56.9%), while 44 (30.6%) had a depressive episode, 12 (8.3%) had a hypomanic episode, 4 (2.8%) had a manic episode, and 2 (1.4%) had a psychotic episode with affective symptoms not meeting the criteria for any major mood episode.

First-degree family history of psychiatric disorders was reported in 84 (58.3%) patients, with family history of mood disorders being reported in 70 (48.6%) patients and family history of anxiety disorders in 22 (15.3%). First-degree family histories of dementia and Parkinson’s Disease were noted in 30 (20.8%) and 12 (8.3%) patients, respectively.

One-half of the entire sample was affected by PPDs (N = 73, 50.6%) without MBI or MNDs. This group showed an average MBI-C score of 0.32 ± 1.35 with a range from 0 to 7. On the other hand, 40 (27.8%) patients of the entire sample were diagnosed with MBI, with an average MBI-C score of 20.38 ± 8.98, ranging from 9 to 54. The remaining 31 (21.5%) subjects of the entire sample were diagnosed with MNDs, including 10 with MNDs due to multiple etiologies, 6 with AD, 5 with a frontotemporal neurocognitive disorder, 4 with a vascular neurocognitive disorder, and 6 with unspecified MNDs with etiologies under investigation. Overall, 62 (43.1%) patients had vascular leukoencephalopathy based on neuroimaging findings and 34 (23.6%) satisfied criteria for the diagnosis of MCI.

### 3.2. Subgroups Comparisons

No significant differences between patients with PPDs, MBI, and MNDs were found for age, gender, lifetime mood and anxiety disorders, first-degree family history of dementia and Parkinson’s Disease, and depressive symptom severity (Table 1).

Nevertheless, although not reaching statistical significance, bipolar disorder type 1 was more common in patients with PPDs, especially with respect to patients with MNDs (*p* = 0.053, *p*_corr_ = 0.158), while bipolar disorder type 2 was less common in patients with MBI compared to both patients with PPDs (*p* = 0.041, *p*_corr_ = 0.123) and patients with MNDs (*p* = 0.092, *p*_corr_ = 0.137).

There were also no differences among groups for mood state at the time of the assessment, with the exception of psychotic episodes with affective symptoms not meeting the criteria for any major mood episode (*p* = 0.045). Indeed, psychotic episodes were diagnosed in two patients with MNDs and in none of the patients with PPDs (*p* = 0.087, *p*_corr_ = 0.260) or MBI (*p* = 0.187, *p*_corr_ = 0.280). Moreover, an almost significant difference was also found for manic episodes (*p* = 0.076), since no current manic episode was diagnosed in patients with PPDs, while two patients with MBI (*p* = 0.123, *p*_corr_ = 0.184) and two patients with MNDs (*p* = 0.087, *p*_corr_ = 0.184) satisfied the criteria for mania at the time of the assessment.

Age at onset of psychiatric symptoms significantly differed among the groups, with a significantly earlier onset in patients with PPDs compared to both those with MBI (*p* = 0.015, *p*_corr_ = 0.046) and MNDs (*p* = 0.025, *p*_corr_ = 0.037). Age at onset of depression also differed among the groups, with a significantly later onset in patients with MBI compared to those with PPDs (*p* = 0.003, *p*_corr_ = 0.009). On the other hand, age at onset of (hypo)manic episodes was significantly lower in patients with PPDs compared to those with MNDs (*p* = 0.009, *p*_corr_ = 0.026), and, although non-significantly, to those with MBI (*p* = 0.046, *p*_corr_ = 0.070). Overall, differences in age at onset of mood disorders did not reach the significance threshold (*p* = 0.075). However, a trend toward the earlier onset of mood disorders in patients with PPDs compared to those with MNDs was also observed (*p* = 0.043, *p*_corr_ = 0.130), with no significant difference with respect to MBI (*p* = 0.109, *p*_corr_ = 0.164).

First-degree family history of psychiatric disorders significantly differed among the groups. Patients with PPDs showed higher familiarity with psychiatric disorders compared to those with MNDs (*p* = 0.008, *p*_corr_ = 0.024), whereas MBI patients showed an intermediate rate among the groups. A similar but less statistically significant pattern was observed for first-degree family history of mood (*p* = 0.054) and anxiety disorders (*p* = 0.094), with more pronounced differences observed between patients with PPDs and MNDs (respectively, *p* = 0.031, *p*_corr_ = 0.094; *p* = 0.087, *p*_corr_ = 0.195).

Similarly, vascular leukoencephalopathy also significantly differed between the groups, since it was significantly less prevalent in patients with PPDs compared to those with MNDs (*p* = 0.028, *p*_corr_ = 0.084) and, although non-significantly, to those with MBI (*p* = 0.106, *p*_corr_ = 0.159). As expected, MCI was significantly more prevalent in patients with MBI compared to those with PPDs. MMSE total score, instead, was significantly lower in patients with MNDs compared to both the other groups (*p* < 0.001, *p*_corr_ < 0.001), but did not distinguish between PPDs and MBI (*p* = 0.975, *p*_corr_ = 0.975).

Psychopathology according to CGI-S was significantly greater in patients with MNDs compared to both the other groups (*p* < 0.001, *p*_corr_ < 0.001), while no significant differences occurred between PPDs and MBI (*p* = 0.603, *p*_corr_ = 0.603). Similarly, patients with MNDs had significantly greater impairment of functioning compared to both PPDs and MBI (*p* < 0.001, *p*_corr_ < 0.001). Conversely, no difference in functioning was found between these latter groups (*p* = 0.171, *p*_corr_ = 0.171). The BPRS total score and Negative Symptoms/Retardation subscale instead significantly differed between each pair of groups with greater symptom severity in patients with MNDs (vs. PPDs: respectively, *p* < 0.001, *p*_corr_ < 0.001 and *p* < 0.001, *p*_corr_ < 0.001; vs. MBI: respectively, *p* = 0.002, *p*_corr_ = 0.002 and *p* < 0.001, *p*_corr_ < 0.001), intermediate severity in those with MBI, and lower severity in patients with PPDs (vs. MBI: respectively, *p* = 0.026, *p*_corr_ = 0.026 and *p* = 0.017, *p*_corr_ = 0.017). Moreover, patients with MNDs scored significantly higher than those with PPDs (*p* < 0.001, *p*_corr_ = 0.002) and almost significantly higher than those with MBI (*p* = 0.057, *p*_corr_ = 0.086) on the BPRS Activation subscale. Similarly, patients with MNDs scored significantly higher on the BPRS Psychosis subscale than both those with PPDs (*p* < 0.001, *p*_corr_ < 0.001) and those with MBI (*p* = 0.002, *p*_corr_ = 0.003).

## 4. Discussion

In this study, we systematically evaluated the occurrence of MBI in a sample of 144 individuals aged ≥50 years referred to our psychogeriatric outpatient service for mental health care. If excluding patients with MNDs or neurodegenerative Parkinsonism, more than one-third of subjects referred to our psychogeriatric service satisfied the criteria for MBI (N = 40/113, 35.4%). This result is in line with a recent systematic review and meta-analysis of studies reporting a prevalence of 33.5% in patients aged ≥50 years with and without cognitive decline [53]. According to the review, MBI prevalence increases along with cognitive deterioration, with the highest prevalence observed in patients with MCI (45.5%), followed by those with subjective cognitive impairment or who are cognitively normal but at risk (35.8%) and, finally, by cognitively normal subjects (17.0%).

Importantly, in our sample, formal psychiatric diagnoses of mood and anxiety disorders were equally represented both in patients with MBI and MNDs with respect to those with PPDs, highlighting that MBI may often co-occur with common psychiatric conditions in psychogeriatric samples. Accordingly, a diagnosis of MBI should not be ruled out solely based on the presence of psychiatric disorders meeting traditional diagnostic criteria. No difference was also found for depressive symptom severity, confirming our previous findings showing a common overlap between major depression and MBI [40].

On the other hand, several differences between the groups supported the validity of the MBI construct and underlined the potential hallmarks of this condition. First, the age at onset of psychiatric symptoms was similarly higher, by approximately 10 years, in patients with MBI and MNDs compared to those with PPDs. Particularly, the onset of depression in patients with MBI manifested, on average, 15 years later than patients with PPDs. Depression first occurring in late life has been closely associated with cognitive decline and negative outcomes, including progressive dementia and AD [54,55]. More recently, a cohort study found that affective symptoms meeting MBI criteria had a significantly greater progression rate to dementia than affective symptoms not meeting MBI criteria and potentially meeting DSM criteria for depression [56]. Manic syndromes may also occur, albeit less often, in the context of neurodegeneration [57]. Accordingly, in our sample, manic episodes were diagnosed at the time of assessment only in patients with MNDs and MBI.

Unexpectedly, no significant differences between patients with PPDs, MBI, and MNDs were found for first-degree family history of dementia and Parkinson’s Disease. Conversely, patients with MBI showed rates of first-degree family history of psychiatric disorders that were intermediate between those of patients with PPDs and those of patients with MNDs. Previous studies found positive family history and early age at onset of psychiatric disorders to be related, suggesting a low genetic load in late-onset conditions [58,59,60,61]. We may hypothesize that MBI is a genetically heterogeneous condition, where a moderate genetic load for psychiatric disorders may not be sufficient to give rise to behavioral symptoms up until acquired neurodegenerative changes occur.

Further support for the putative neurodegenerative nature of MBI was given by the higher prevalence of MCI and, although non-significantly, of vascular leukoencephalopathy in patients with MBI compared to those with PPDs. A statistically significant association between the MBI diagnosis and white matter hyperintensity (WMH) volume has been reported in patients with MCI. Miao and colleagues, in fact, found that MCI patients with MBI had 9.4% more WMH volume than those without MBI [62]. While the presence of MBI was significantly associated with lower MMSE scores in patients with MCI [62], in our sample, the MMSE total score did not differentiate MBI from PPDs. Psychiatric conditions, especially during late life, may cause cognitive impairments that are sometimes not distinguishable from those observed in neurodegenerative disorders, although they show a reversible course [63]. Accordingly, MMSE alone should not be considered a reliable proxy of progressive cognitive decline, even in the presence of MBI, especially given that it was developed to identify dementia, not pre-dementia syndromes.

Higher severity of mental illness, more severe psychopathology and lower global functioning were found in patients with MNDs compared to the other groups. Conversely, no differences in global severity of illness and functioning were found between MBI and PPDs. These latter results mirror those observed in our previous analyses on depressed patients. Nonetheless, despite similar functional impairments at baseline, MBI was associated with a lack of functional recovery after treatment [40]. Consequently, in order to diagnose MBI, longitudinal trajectories of functioning rather than cross-sectional evaluations of functional impairments should be taken into account.

Finally, qualitative differences between patients with MBI and PPDs emerged with respect to psychopathology. Indeed, patients with MBI showed significantly higher scores than those with PPDs on BPRS Negative Symptoms/Retardation, including items related to apathy, such as blunted affect, emotional withdrawal, and uncooperativeness. Apathy is a neuropsychiatric transnosographic syndrome characterized by cognitive, emotional, and behavioral manifestations [64]. According to research, apathy has been associated with cognitive decline. Apathy, in fact, is frequently found in prodromal phases of AD and has been linked to the evolution of cognitive abnormalities throughout the neurodegenerative continuum [65,66,67,68]. In particular, apathy, but not depression, predicts the progression to AD in patients with amnestic MCI [65]. Although it is unclear whether apathy is a causal factor of cognitive impairment, an epiphenomenon, or a result of neurodegeneration, its presence may serve as a red flag for the diagnosis of MBI. Recently, a study comparing MBI apathy, apathy not meeting MBI criteria, and a group with no NPSs demonstrated a greater rate of incident dementia in the MBI apathy group [69]. These findings further highlight the importance of the apathy syndrome, especially when emerging in late life, for dementia prognostication.

The accumulated data have linked MBI symptoms to the presence of biomarkers of neurodegeneration [37,38,39]. However, more studies are required to confirm this association. In addition to the already studied biomarkers, it would be important to investigate the relationship between MBI symptoms and functional imaging changes. Moreover, it would be interesting to elucidate whether biomarkers of neurodegeneration can distinguish patients with MBI from those with PPDs.

Some limitations of our study should be acknowledged. First, the clinical setting (a tertiary psychiatric unit) may not have been representative of all elderly patients with psychiatric conditions or behavioral symptoms, but rather of a subpopulation at higher risk for a more complicated course, possibly showing a higher prevalence of MBI. Moreover, the relatively limited sample size may reduce the generalizability of the results, which need replication. In addition, several comparisons were performed without controlling for multiple comparisons, given the exploratory nature of the analyses. Future studies from independent samples are, thus, required to corroborate our preliminary findings.

In conclusion, given the risk of cognitive decline associated with MBI, its recognition may help to establish the prognosis of late-life behavioral disorders, plan proper clinical management, and develop new treatments to delay neurodegenerative processes. In our study, MBI was found to be a common condition in patients aged ≥50 years referred to psychogeriatric services, affecting approximately one-third of subjects without MNDs. MBI often co-occurred with psychiatric conditions such as mood and anxiety disorders. However, a higher age at the onset of psychiatric symptoms, particularly of depression, and a higher prevalence of MCI characterized patients with MBI with respect to PPDs. Despite no significant differences in global severity of illness, depressive symptom severity and overall functioning, MBI patients showed greater motor retardation and apathy compared to those with PPDs. These features may help to individuate patients with MBI in psychogeriatric settings.

## Figures and Tables

**Table 1:** Differences between patients with primary psychiatric disorders (PPDs), Mild Behavioral Impairment (MBI) and major neurocognitive disorders (MND).

	PPDs (N = 73)	MBI (N = 40)	MNDs (N = 31)	Stat	*p*	Post Hoc
**Demographic variables**						
Gender (male)	25 (34.2%)	19 (47.5%)	10 (32.3%)	2.40	0.301	-
Age (years)	74.05 ± 8.45	74.72 ± 7.4	76.06 ± 8.07	1.38	0.503	-
**Mood disorders**				16.57	0.167	-
Any mood disorder	60 (82.2%)	30 (75%)	22 (71%)	1.83	0.400	-
Major depressive disorder (recurrent)	7 (9.59%)	6 (15%)	1 (3.23%)		0.255	-
Major depressive disorder (single episode)	7 (9.59%)	9 (22.5%)	7 (22.58%)		0.101	-
Bipolar disorder 1	17 (23.29%)	5 (12.5%)	2 (6.45%)		0.082	-
Bipolar disorder 2	18 (24.66%)	3 (7.5%)	7 (22.58%)		0.071	-
Cyclothymic disorder	6 (8.22%)	3 (7.5%)	2 (6.45%)		1.000	-
Bipolar disorder NOS	5 (6.85%)	4 (10%)	3 (9.68%)		0.785	-
Without mood disorders	13 (17.81%)	10 (25%)	9 (29.03%)		0.406	-
**Anxiety disorders**						
Any anxiety disorder	45 (61.6%)	18 (45.0%)	18 (58.1%)	2.96	0.228	-
Panic disorder	32 (43.8%)	10 (25%)	14 (45.2%)	4.51	0.105	-
Agoraphobia	8 (11%)	1 (2.5%)	1 (3.2%)	-	0.212	-
Social anxiety	3 (4.1%)	0 (0%)	0 (0%)	-	0.431	-
Generalized anxiety	23 (31.5%)	10 (25%)	8 (25.8%)	0.68	0.714	-
**Current state**				13.11	0.108	-
Depressive episode	26 (35.62%)	11 (27.5%)	7 (22.58%)		0.376	-
Hypomanic episode	6 (8.22%)	3 (7.5%)	3 (9.68%)		1.000	-
Manic episode	0 (0%)	2 (5%)	2 (6.45%)		0.076	-
Psychotic episode	0 (0%)	0 (0%)	2 (6.45%)		0.045	-
Euthymia	41 (56.16%)	24 (60%)	17 (54.84%)	0.23	0.893	-
**Age at onset**						
Age at onset of psychiatric symptoms (N = 140)	41.48 ± 24.12	52.85 ± 22.02	53.45 ± 20.92	8.20	0.017	MNDs, MBI > PPDs
Age at onset of mood disorders (N = 112)	42.77 ± 21.6	50.53 ± 20.66	53.55 ± 17.56	5.17	0.075	-
Age at onset of depression (N = 70)	42.08 ± 20.31	57.27 ± 17.65	49.2 ± 14.37	8.82	0.012	MBI > PPDs
Age at onset of (hypo)mania (N = 39)	45.46 ± 18.83	58.75 ± 19.21	66.57 ± 9.4	8.88	0.012	MNDs > PPDs
**First-degree family history**						
Dementia	14 (19.2%)	8 (20%)	8 (25.8%)	0.60	0.740	-
Parkinson’s disease	7 (9.6%)	2 (5%)	3 (9.7%)	-	0.725	-
Psychiatric disorders	50 (68.5%)	22 (55%)	12 (38.7%)	8.19	0.017	PPDs > MNDs
Mood disorders	42 (57.5%)	18 (45%)	10 (32.3%)	5.85	0.054	-
Anxiety disorders	16 (21.9%)	4 (10%)	2 (6.5%)	-	0.094	-
**Vascular leukoencephalopathy**	24 (32.9%)	20 (50%)	18 (58.1%)	6.72	0.035	-
**MCI**	14 (19.2%)	20 (50%)	-	10.25	0.001	-
**MMSE score** (N = 135)	26.45 ± 3.54	26.26 ± 4.49	18.64 ± 8.56	20.25	0.000	PPDs, MBI > MNDs
**Psychometric assessment**						
CGI—Severity of illness	3.03 ± 1.17	3.2 ± 1.07	4.23 ± 1.09	21.32	0.000	MNDs > PPDs, MBI
Global Assessment of Functioning	67.05 ± 16.93	61.62 ± 17.15	36.61 ± 14.57	45.36	0.000	PPDs, MBI > MNDs
BPRS total score	35.79 ± 7.96	40.35 ± 10.01	50.71 ± 13.35	32.31	0.000	MNDs > MBI > PPDs
BPRS Depression/Anxiety	12.34 ± 3.68	12.35 ± 3.72	13.45 ± 4.59	1.09	0.580	-
BPRS Activation	12.75 ± 4.36	15.45 ± 7.67	17.74 ± 7.51	11.90	0.003	MNDs > PPDs
BPRS Negative Symptoms/Retardation	7.03 ± 2.72	8.35 ± 3.32	12.52 ± 5.05	36.33	0.000	MNDs > MBI > PPDs
BPRS Psychosis	7.3 ± 2.06	8.43 ± 4.04	12.13 ± 6.75	19.95	0.000	MNDs > PPDs, MBI

PPDs: Primary psychiatric disorders; MBI: mild behavioral impairment; MNDs: major neurocognitive disorders; NOS: not otherwise specified; MCI: mild cognitive impairment; MMSE: Mini-Mental State Examination; CGI: Clinical Global Impression; BPRS: Brief Psychiatric Rating Scale.

## Data Availability

The data that support the findings of this study are available upon reasonable request from the corresponding author.
